# Progression to type 2 diabetes mellitus after gestational diabetes mellitus diagnosed by IADPSG criteria: Systematic review and meta-analysis

**DOI:** 10.3389/fendo.2022.1012244

**Published:** 2022-10-06

**Authors:** Juan Juan, Yiying Sun, Yumei Wei, Shuang Wang, Geng Song, Jie Yan, Pengxiang Zhou, Huixia Yang

**Affiliations:** ^1^ Department of Obstetrics and Gynecology, Peking University First Hospital, Beijing, China; ^2^ Department of Obstetrics and Gynecology, Peking Union Medical College Hospital, Peking Union Medical College, Chinese Academy of Medical Sciences, Beijing, China; ^3^ Department of Pharmacy, Peking University Third Hospital, Beijing, China; ^4^ Institute for drug evaluation, Peking University Health Science Center, Beijing, China

**Keywords:** gestational diabetes mellitus, type 2 diabetes mellitus, IADPSG criteria, systematic review, meta-analysis

## Abstract

**Background:**

To estimate the progression rates to type 2 diabetes mellitus (T2DM) in women with gestational diabetes mellitus (GDM) diagnosed by the International Association of Diabetes and Pregnancy Study Group (IADPSG) criteria.

**Methods:**

Systematic review and meta-analysis were conducted by searching Medline, Embase, and Cochrane between January 1, 2010 and December 31, 2021 for observational studies investigating progression to T2DM after GDM. Inclusion criteria were IADPSG-diagnosed GDM, studies with both GDM and controls, postpartum follow-up duration at least one year. Data were pooled by random effects meta-analysis models. Heterogeneity was assessed by I_2_ statistic. The pooled relative risk for incidence of T2DM and pre-diabetes between GDM participants and controls were estimated. Reasons for heterogeneity among studies were investigated by prespecified subgroup and meta-regression analysis. Publication bias was assessed by the Begg’s and Egger’s tests.

**Results:**

This meta-analysis of six studies assessed a total of 61932 individuals (21978 women with GDM and 39954 controls). Women with IADPSG-diagnosed GDM were 6.43 times (RR=6.43, 95% CI:3.45-11.96) more likely to develop T2DM in the future compared with controls. For GDM women, the cumulative incidence of T2DM was 12.1% (95% CI: 6.9%-17.3%), while the pooled cumulative incidence of T2DM was estimated to be 8% (95% CI: 5-11%) in studies with 1 to 5 years of follow-up and increased to 19% (95% CI: 3-34%) for studies with more than 5 years of follow-up. Women with IADPSG-diagnosed GDM had 3.69 times (RR=3.69, 95% CI:2.70-5.06) higher risk of developing pre-diabetes (including impaired fasting glucose and/or impaired glucose tolerance) than controls. Meta-regression analysis showed that the study effect size was not significantly associated with study design, race, length of follow-up, and maternal age (P>0.05). Overall, the studies had a relatively low risk of bias.

**Conclusions:**

Women with IADPSG-diagnosed GDM have higher risk of developing T2DM and pre-diabetes. The risk of T2DM in GDM women are higher with longer follow-up duration. Our results highlight the importance of promoting postpartum screening and keeping health lifestyle as well as pharmacological interventions to delay/prevent the onset of T2DM/pre-diabetes in GDM women.

**Systematic review registration:**

https://www.crd.york.ac.uk/prospero, identifier (CRD42022314776)

## Introduction

Gestational diabetes mellitus (GDM) is an established risk factor for developing type 2 diabetes mellitus (T2DM) in later life. Previous meta-analysis has highlighted a nearly 10-fold higher risk of T2DM in women with GDM compared with controls (1). In 2010, the International Association of Diabetes and Pregnancy Study Group (IADPSG) proposed new diagnostic criteria for GDM using a one-step strategy with 75g oral glucose tolerance test (OGTT) during 24–28 weeks of gestation, and the diagnostic thresholds for GDM were identified as one or more of plasma glucose values equaling or exceeding 5.1 mmol/L (92 mg/dl), 10.0 mmol/L (180 mg/dl), and 8.5 mmol/L (153 mg/dl) at fasting, 1-hour, and 2-hour after 75g OGTT (2). This was the first evidence-based, large-scale diagnostic criteria based on glucose levels associated with adverse pregnancy outcomes in the Hyperglycemia and Adverse Pregnancy Outcomes (HAPO) Study (3). The IADPSG diagnostic criteria marked a milestone in the history of GDM diagnostic criteria, and many international organizations, including American Diabetes Association (ADA) (4), World Health Organization (WHO) (5), the International Federation of Gynecology and Obstetrics (6) et al. advocated the use of 75g OGTT during 24–28 weeks of gestation as the diagnostic test and the new cutoff values recommended by IADPSG as GDM diagnostic criteria.

The application of this landmark IADPSG diagnostic criteria led to a rise in the prevalence of GDM globally, which contribute to a rise in the healthcare and economic burden worldwide (7–10). Due to changes in GDM diagnosis criteria, although the IADPSG criteria had been implemented for almost 12 year, previous systematic review and meta-analysis evaluating progression to T2DM after GDM did not distinguish different kinds of diagnosis criteria, and no available meta-analysis reported the risk of T2DM and pre-diabetes after GDM diagnosed by IADPSG criteria. As is well-known, lifestyle and pharmacological intervention could prevent or delay progression to T2DM in women with previous GDM (11). Therefore, there is an urgent need to evaluate more recent evidence on the risk of progression to T2DM in women with GDM diagnosed by IADPSG criteria. This systematic review and meta-analysis aimed to investigate progression to T2DM in women with GDM diagnosed by IADPSG criteria compared with controls.

## Methods

This systematic review and meta-analysis were conducted in accordance with the Preferred Reporting Items for Systematic Reviews and Meta-Analyses (PRISMA) and Meta-Analyses Of Observational Studies in Epidemiology (MOOSE) guidelines. The protocol of the study had been registered on the International prospective register of systematic reviews (PROSPERO) (registration number: CRD42022314776).

### Search strategy

We conducted a comprehensive literature search using Medline, Embase, the Cochrane Library, Scopus, AJOL, and Hinari for observational studies investigating progression to T2DM after GDM diagnosed by IADPSG criteria published between January 1, 2010 and December 31, 2021. We chose 2010 as the start date because the IADPSG GDM diagnostic criteria was released in 2010. The search strategy included keywords, medical subject headings (MeSH), and free text words covering “gestational diabetes mellitus” and “type 2 diabetes mellitus”, and was restricted to studies published in English and conducted on humans. The search strategy was shown in the **Supplementary Material**.

### Study selection

**Figure 1:**
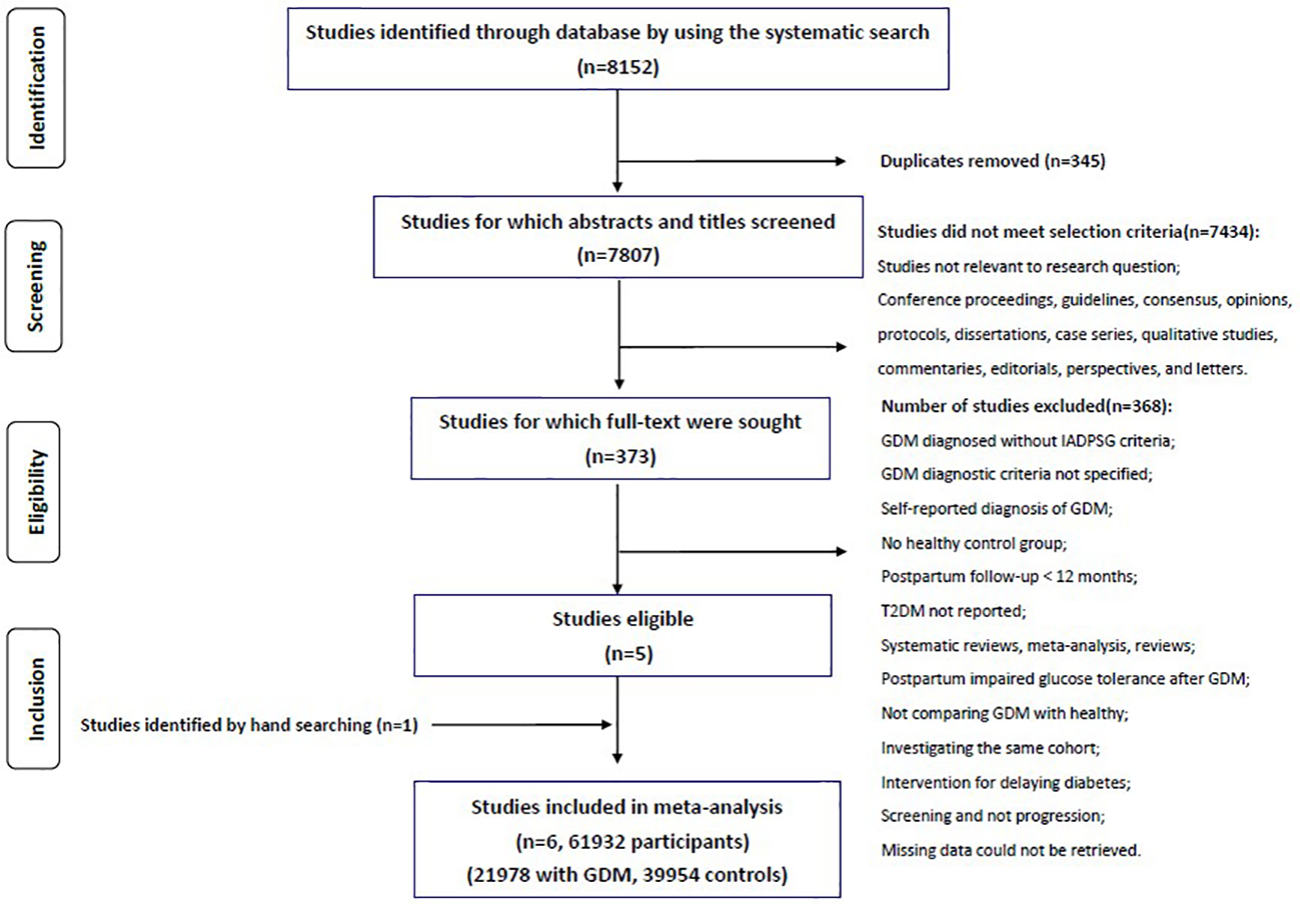
PRISMA flowchart showing literature search and study inclusion of the meta-analysis.

Two authors (J.J. and YY.S.) independently reviewed the titles and abstracts to identify all potentially relevant studies. All duplicate records were removed. Full text of the relevant studies were obtained and screened in details according to the following predefined eligibility criteria: GDM diagnosed by IADPSG criteria, duration of postpartum follow-up at least one year, studies with both GDM women and controls (pregnant women without GDM). All reference lists from relevant reviews were hand searched for additional eligible studies. All conference proceedings, guidelines, consensus, opinions, protocols, dissertations, case series, qualitative studies, commentaries, editorials, perspectives, and letters were excluded. Disagreements between the two authors were resolved by third party consultation. The literature review and study selection process were summarized in a PRISMA flowchart (**Figure 1**).

### Data extraction

Two authors (J.J. and YY.S.) independently extracted information from the included studies according to the Cochrane Handbook guidelines, and findings were reported according to the PRISMA and MOOSE guidelines. The extracted data was exported to an excel sheet detailing the authors’ name, country of origin of the study, year of publication, study design, sample size (including the number of GDM and control), length of follow-up, participants’ age, race, diagnostic criteria for T2DM, incidence of T2DM and pre-diabetes (including impaired fasting glucose[IFG] and/or impaired glucose tolerance[IGT]) among GDM and control, etc. Disagreements were settled by consensus among all authors.

### Study quality assessment and risk of bias

The Newcastle-Ottawa scale (NOS) was used to evaluate the quality of non-randomized studies (12). The scale for cohort studies consisted of three categories: Selection, Comparability, and Outcome. Based on the guideline of the scale, a cohort study could be awarded a maximum of one star for each item of the Selection and Outcome categories and a maximum of two stars for the category of comparability. Totally, a study could be awarded from zero up to nine stars. Publication bias was assessed by the Begg’s and Egger’s tests and funnel plots diagram for asymmetry.

### Statistical analysis

The pooled relative risk for the incidence of T2DM and pre-diabetes between participants with GDM and controls was estimated. Data were pooled by random effects meta-analysis models with the Review Manager (version 5.4; Nordic Cochrane Centre, Cochrane Collaboration, Copenhagen, Denmark) using the DerSimonian-Laird method. In studies which no cases of T2DM and pre-diabetes was reported in any of the groups, a continuity correction was applied using the default value of 0.5 to avoid division by zero. Heterogeneity among the included studies was assessed by I^2^ statistic, and graphically represented using forest plot diagram. Pre-specified subgroup analyses by race and length of study follow-up was performed to explore potential sources of heterogeneity among studies. Meta-regression models were fitted to estimate the effects of study heterogeneity and investigated the cumulative risk of developing T2DM by study length of follow-up. Sensitivity analysis were conducted by recalculating the pooled estimate with a named study removed at a time to estimate the effect of each individual study on the overall pooled estimate.

## Results

### Study selection

The initial comprehensive literature search identified 8152 records. After exclusion of duplicates, 7807 articles were screened, of which 373 studies were selected for further detailed review using the full text. One study retrieved from the reference lists of previous relevant reviews were also included. Finally, six studies, including a total of 61932 individuals (21978 women with GDM and 39954 controls) fulfilled all the eligibility criteria and were included in the meta-analysis. The study selection process was summarized in **Figure 1**.

### Study quality assessment

All six included studies underwent quality assessment with NOS scale received a total of six to eight stars, suggesting that the risk of bias is relatively low. A summary of the study quality assessment was shown in the **Supplementary Material-STable 1**.

### Study characteristics

All studies included in this systematic review and meta-analysis were observational studies, of which three were prospective cohorts and the other three were retrospective cohorts. One study was a multi-centered study including ten field centers (13), the rest five studies were conducted in Canada, Australia, United Arab Emirates, Pakistan, and Japan, respectively (14–18). The six included studies had different lengths of follow-up, ranged from 1.3 to 11.4 years. A summary of study characteristics of the included studies was presented in **Table 1**.

**Table 1:** Study characteristics.

Author	Year	Country	Study design	Paticipants’ age	Race	GDM sample size	Control Sample Size	Total Sample Size	Follow-up (years)	T2DM diagnostic criteria	T2DM/GDM	T2DM/Control	Pre-diabetes/GDM	Pre-diabetes/Control
Hiersch et al. (12)	2021	Canada	retrospective cohort	30.55	Mixed	20513	34848	55361	4.4	Diabetes Canada 2018 clinical practice guidelines	1132/20513	244/34848	–	–
Wood et al. (13)	2021	Australia	prospective cohort	GDM 29; control 25	White	172	122	294	2.5	WHO, ADA	11/172	2/122	16/172	1/122
Bayoumi et al. (14)	2021	United Arab Emirates	retrospective cohort	38.7	Non-white	362	833	1195	9	ADA	96/362	9/833	171/362	145/833
Aziz et al. (15)	2018	Pakistan	prospective cohort	GDM 28.94; control 25.68	Non-white	78	89	167	2	HbA1C: ≥6.5%; or FBG: ≥126 mg/dL (7.0 mmol/L); or 2-h blood glucose: ≥200 mg/dL (11.1 mmol/L) during an OGTT; or A random plasma glucose: ≥200 mg/dL (11.1 mmol/L)	11/78	0/89	3/78	–
Lowe et al. (11)	2018	Multi-country including 10 field centers	prospective cohort	GDM 31.9; control 29.8	Mixed	663	3946	4609	11.4	ADA	71/663	63/3946	275/663	728/3946
Kugishima et al. (16)	2018	Japan	retrospective cohort	GDM 32.9; control 33.2	Non-white	190	116	306	1.3	WHO	21/190	11/116	–	–

### Meta-analysis

**Figure 2:**
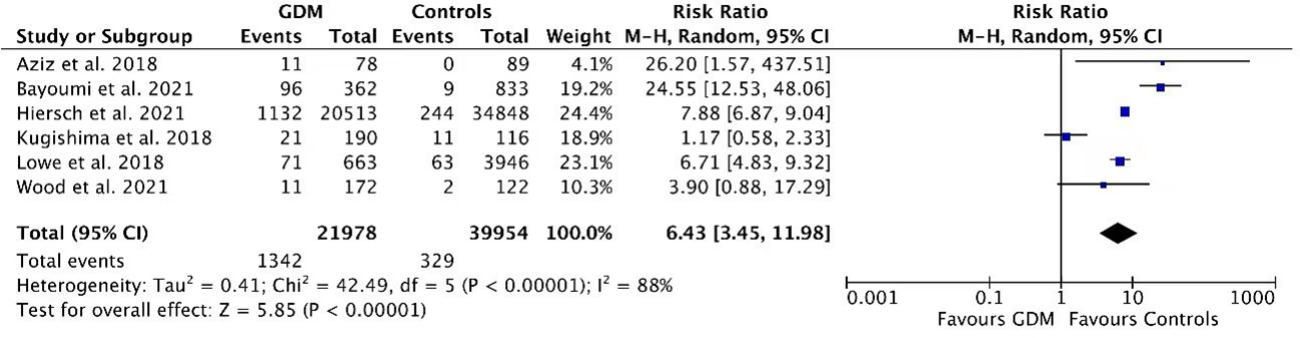
Relative risk of T2DM in women with GDM compared with controls.

**Figure 3:**
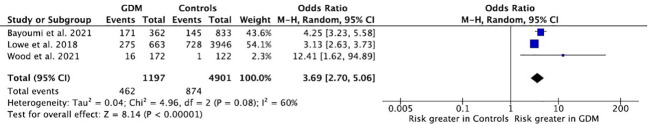
Relative risk of pre-diabetes in women with GDM compared with controls.

The meta-analysis of the six studies evaluated a total of 61932 participants (21978 women with GDM and 39954 controls). Among the participants, 1342 women from the GDM group subsequently developed T2DM during postpartum follow-up, while 329 women progressed to T2DM in the control group. The pooled relative risk of developing T2DM in the GDM group was 6.43 (RR=6.43, 95% CI:3.45-11.96) compared with controls (**Figure 2**). Significant heterogeneity was seen in the overall effect estimate (I2 = 88.2%, P<0.001) (**Figure 2**). In addition, women who had GDM were 3.69 times (RR=3.69, 95% CI:2.70-5.06) more likely to develop pre-diabetes (including IFG and/or IGT) than controls (**Figure 3**).

**Figure 4:**
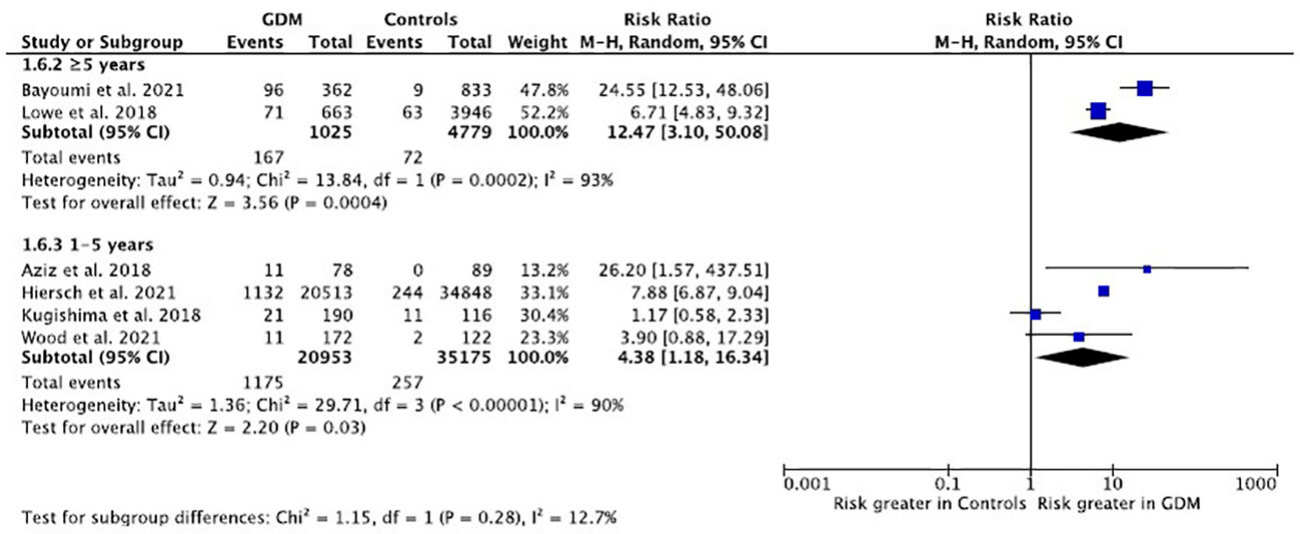
Relative risk of T2DM in women with GDM compared with controls based on study length of follow-up.

The risk of T2DM were assessed by pre-specified subgroup analysis based on study length of follow-up. Studies were classified by their length of follow-up into two groups: 1-5 years and more than 5 years. The estimated pooled relative risk of developing T2DM was 4.38 (95% CI:1.18-16.34) for studies with postpartum follow-up of 1-5 years, while for those with follow-up of more than five years, the pooled relative risk was 12.47 (95% CI:3.10-50.08). The pooled relative risks were not statistically significantly different between subgroups (P=0.28) (**Figure 4**). The relative risk of T2DM was also assessed by subgroup analysis based on race and the difference in pooled relative risks by subgroup was not statistically significant (P=0.67).

Further meta-regression analyses showed that the study effect size was not significantly associated with study design, race, length of follow-up, and maternal age (P> ˃0.05) (**Table 2**). Overall, in women with GDM, the cumulative incidence of T2DM and pre-diabetes were 12.1% (95% CI: 6.9%-17.3%) and 25.5% (95% CI: 4.1%-46.8%), respectively. The pooled cumulative incidence of T2DM was estimated to be 8% (95% CI: 5%-11%) in women with GDM in studies with 1 to 5 years of follow-up, increasing to 19% (95% CI: 3%-34%) for studies with more than 5 years of follow-up (**Table 3**).

**Table 2:** Results of meta-regression models.

Study level variables	Coefficient (95%CI)	P values	I^2^ (%)
Study design			
Retrospective cohort study	0.84 (0.04, 16.90)	0.884	90.37
Prospective cohort study	Ref.	
Race			
White	1.01 (0.02, 66.58)	0.992	92.55
Non-white	0.54 (0.00,185.31)	0.757	92.55
Mixed	Ref.	
Length of follow-up (mean)	1.51 (0.84, 1.57)	0.277	89.95
Age (mean)	1.08 (0.75, 1.55)	0.589	89.58

**Table 3:** Cumulative incidence of T2DM by length of follow-up.

	No of contributing studies	GDM (%; 95% CI)	Controls (%; 95% CI)
Length of follow-up (years):			
1-5	4	0.08 (0.05, 0.11)	0.02 (0.00, 0.03)
≥5	2	0.19 (0.03, 0.34)	0.01 (0.01, 0.02)
Overall	6	0.12 (0.07, 0.17)	0.01 (0.005,0.02)

### Publication bias and sensitivity analysis

No indication of publication bias was detected among the included studies, with both Begg’s and Egger’s tests being statistically non-significant (P=0.71 and P=0.76, respectively). No apparent asymmetry was observed in the funnel plots diagram of the six studies included in the meta-analysis (**Supplementary Material-SFigure 1**).

Sensitivity analysis was conducted, in which a named study was removed, and the others were analyzed, the pooled estimate remained close to the overall estimate and without apparent fluctuation, indicating that no individual study had a large influence on pooled estimate.

## Discussion

### Main findings

The results of the systematic review and meta-analysis demonstrated that women who had GDM diagnosed by IADPSG criteria were 6.43 times (RR=6.43, 95% CI:3.45-11.96) more likely to develop T2DM in the future compared with controls. In women with previous GDM, the cumulative incidence of T2DM was 12.1% (95% CI: 6.9%-17.3%), while the pooled cumulative incidence of T2DM was estimated to be 8% (95% CI: 5-11%) in studies with 1 to 5 years of follow-up and increased to 19% (95% CI: 3-34%) for studies with more than 5 years of follow-up. Women with IADPSG-diagnosed GDM had 3.69 times (RR=3.69, 95% CI:2.70-5.06) higher risk of developing pre-diabetes than controls. Meta-regression analysis showed that the study effect size was not significantly associated with study design, race, length of follow-up, and maternal age (*P*>0.05). Overall, the studies had a relatively low risk of bias.

### Comparison with previous studies

In this systematic review and meta-analysis, we included evidence from the most recent studies and specified GDM diagnostic criteria to IADPSG to estimate the risk of developing T2DM after GDM. The findings of our study suggested a higher risk of T2DM and pre-diabetes in women with GDM than controls, similar trends reported by previous systematic reviews and meta-analysis (1, 19, 20). However, previous studies conducted by Kim et al. published in Diabetes Care in 2002 (20), Bellamy et al. published in the Lancet in 2009 (19), and Vounzoulaki et al. published in the BMJ in 2020 (1) evaluated studies published between 1965-2001, 1960-2009 and 2000-2020, respectively, which involved GDM women diagnosed by different diagnostic criteria, thus could have led to different overall risk of subsequent T2DM in women with GDM. The systematic review and meta-analysis conducted by Bellamy et al. (19) and Vounzoulaki et al. (1) reported a 7.43 times (RR=7.43, 95% CI:4.79-11.51) and 9.51 times (RR=9.51, 95% CI:7.14-12.67) higher risk of developing T2DM in women with previous GDM than controls, respectively, which was higher than our findings. The systematic review conducted by Kim et al. (20) demonstrated that the cumulative incidence of T2DM increased markedly in the first 5 years after delivery and appeared to plateau after 10 years, which was inconsistent with our results that the risk of T2DM was higher in studies with more than 5 years of follow-up than 1 to 5 years in women with previous GDM.

As the definition of GDM in studies conducted by previous systematic reviews and meta-analysis was all hyperglycemia first detected at any time during pregnancy, which also included women with pre-existing diabetes mellitus who were not identified prior to pregnancy and did not distinguish between diabetes in pregnancy and GDM. Women with pre-gestational diabetes mellitus indicated the underlying T2DM short-term after delivery. Therefore, the previous study found that the incidence of T2DM increased markedly in the first 5 years after delivery. Unlike previous systematic reviews, the definition of GDM we used in this study were diabetes first diagnosed in the second or third trimester of pregnancy that is not clearly overt diabetes prior to gestation (4).

Furthermore, the GDM diagnostic criteria had been constantly evolving during the past few decades, with the diagnostic strategy evolved from “two-step strategy” (GDM was diagnosed by stepwise approach: Step 1: 50g fasting plasma glucose test [GCT]; Step 2: For pregnant women whose 1-hour glucose value of 50g GCT is equal to or greater than 7.8 mmol/L [140 mg/dl], 75g/100g OGTT was conducted) (21, 22) to “one-step strategy” (GDM was diagnosed by 75g OGTT during 24-28 gestational week, and the diagnostic thresholds for GDM were identified as one or more of plasma glucose values equaling or exceeding 5.1 mmol/L [92 mg/dl], 10.0 mmol/L [180 mg/dl], and 8.5 mmol/L [153 mg/dl] at fasting, 1-hour, and 2-hour after 75g OGTT) (2); the cut-off value of fasting plasma glucose (FPG) for GDM evolved from 5.8 mmol/L (National Diabetes Data Group [NDDG], 1979) (21) to 5.3 mmol/L (Carpenter and Coustan, 1982) (22), and then to 5.1 mmol/L (IADPSG, 2010) (2); the diagnostic thresholds evolved from two or more abnormalities (21, 22) to one or more abnormalities (2). The newly released IADPSG criteria is the first scientific based diagnostic criteria predominantly based on HAPO study (3) and marked a milestone in the history of GDM diagnostic criteria. The IADPSG criteria had been advocated by many international organizations, including ADA (4), WHO (5), and the international Federation of Gynecology and Obstetrics (6). Studies had shown that the prevalence of GDM had increased significantly by implementation of IADPSG diagnostic criteria and more pregnant women with milder degrees of gestational hyperglycemia were diagnosed as GDM, which provided an opportunity for earlier detection and effective lifestyle intervention of milder GDM to prevent adverse pregnancy outcomes (7–9). We specified GDM diagnostic criteria to IADPSG in this meta-analysis. Therefore, the previous systematic reviews might have caused a higher risk of developing T2DM after GDM than ours.

Our results indicated that even the milder degrees of gestational hyperglycemia diagnosed by the more stringent IADPSG criteria still had a higher risk of subsequent progression to T2DM and pre-diabetes. Genetic predisposition might be one possible contributor to the progression to T2DM after GDM as increasing evidence have indicated that GDM may share similar genetic susceptibilities with T2DM (23–26). Genome wide association studies (GWAS) and other studies have reported that several genetic variants (such as *MTNR1B, TCF7L2, IGF2BP2, CDKAL1, GCK*) are associated with increased risk of both GDM and T2DM, suggesting that these conditions might have a shared genetic background (27, 28). The genetic predisposition for GDM influences the health outcomes in the perinatal stage and poses a risk for T2DM in later life. Women with genetic variants for GDM and/or T2DM are expected to have a higher risk of postpartum diabetes, but further studies are needed to discover the specific genetic variants associated with postpartum diabetes (29). Pre-diabetes was considered to be a precursor for the development of T2DM and women with pre-diabetes might also indicate the underlying frequency of T2DM. So, we should pay more attention to postpartum follow-up of GDM women and introduce structured postpartum preventive care (30). Almost all guidelines recommended T2DM screening with 75g OGTT at 4-12 weeks postpartum and tested every 1-3 years thereafter for women with GDM. Women with a history of GDM found to have pre-diabetes should receive intensive lifestyle interventions and/or metformin to prevent T2DM (4). Despite the emphasis of these guidelines and the magnitude of T2DM risk after GDM, the postpartum screening rates were relatively low and the importance of postpartum follow-up and intervention had not been adequately addressed (31–35).

Both lifestyle and pharmacological intervention prevent or delay progression to T2DM in women with previous GDM. Results of the prospective Nurses’ Health Study (NHS) showed that subsequent T2DM risk after GDM was significantly lower in women who following healthy eating patterns (36). According to a randomized controlled clinical trial conducted at 27 clinical centers, intensive lifestyle intervention and metformin could reduce progression to diabetes by 35% and 40%, respectively, in women with previous GDM over 10 years of follow-up compared with placebo (37). Therefore, we should highlight the importance of increased awareness in women with GDM the need to attend postpartum screening and motivated them to keep healthy lifestyle, including physical exercise and balanced diet, as well as adopt pharmacological interventions under the guidance of doctors if needed in order to delay or prevent the progression from GDM to T2DM (36–38). In the meanwhile, it is important to cooperate obstetricians, internists, pediatricians, and other healthcare providers to provide support and emphasize the importance of postpartum follow-up of GDM to reduce the future risk of T2DM.

More up-to-date large randomized controlled trials with longer follow-up are needed to investigate the lifestyle and pharmacological intervention strategies to delay or prevent the progression from GDM to T2DM. In addition, further cost-effectiveness studies of these interventions should be conducted to promote the implementation of these interventions.

### Strengths and limitations

This systematic review and meta-analysis assessed the most recently published studies on the risk of progression to T2DM in women with IADPSG-diagnosed GDM, with a relatively large total number of individuals, and postpartum follow-up duration ranging from 1.3 to 11.4 years. As the diagnostic criteria of GDM had changed over the past years, this meta-analysis provided up-to-date results using the specific IADPSG criteria. Nevertheless, several limitations should also be considered. Owing to limited availability of studies evaluating the risk of progression to T2DM in women with GDM diagnosed by IADPSG criteria, only a few studies fulfilled all the eligibility criteria and were included in this meta-analysis, resulting in inclusion of a relatively small sample size. Therefore, we were unable to investigate the progression to T2DM after GDM in certain subgroups, which could have been a cause of heterogeneity among studies. Furthermore, we were unable to identify the main sources of heterogeneity in our analysis, and a more in-depth analysis could have been performed if individual patient level data were available. In addition, the IADPSG criteria was released in 2010 and many of the women might have not been developed T2DM at the time of assessment. Therefore, more high-quality studies with longer and complete follow-up are needed to accurately evaluate the progression to T2DM in women with GDM.

## Conclusions

In conclusion, our systematic review and meta-analysis showed that women with GDM diagnosed by IADPSG criteria had higher risk of developing T2DM and pre-diabetes than controls. The risk of T2DM in women with previous GDM was higher in studies with more than 5 years of follow-up than 1 to 5 years. Our results highlight the importance of promoting postpartum screening and keeping health lifestyle, including physical exercise and balanced diet, as well as pharmacological interventions to delay or prevent the onset of T2DM/pre-diabetes in women with GDM.

## Data availability statement

The original contributions presented in the study are included in the article/**Supplementary Material**. Further inquiries can be directed to the corresponding author.

## Author contributions

HY and JJ conceived and designed the study. JJ and YS conducted the study selection and data extraction. JJ and PZ conducted the study quality assessment and statistical analysis. JJ wrote the manuscript. HY, YW, SW, GS, and JY reviewed and edited the manuscript. All authors contributed to the article and approved the submitted version.

## Funding

This research was funded by the Youth Program of National Natural Science Foundation of China (82003528), the National High Level Hospital Clinical Research Funding (“Star of Outlook” Scientific Research Project of Peking University First Hospital) (22cz020301-4803014), the National Key Research and Development Program of China (2021YFC2700700).

## Conflict of interest

The authors declare that the research was conducted in the absence of any commercial or financial relationships that could be construed as a potential conflict of interest.

## Publisher’s note

All claims expressed in this article are solely those of the authors and do not necessarily represent those of their affiliated organizations, or those of the publisher, the editors and the reviewers. Any product that may be evaluated in this article, or claim that may be made by its manufacturer, is not guaranteed or endorsed by the publisher.
