# Supplementary material for: Progression to type 2 diabetes mellitus after gestational diabetes mellitus diagnosed by IADPSG criteria: Systematic review and meta-analysis
Source: Front Endocrinol (Lausanne). 2022 Oct 6;13:1012244. doi: 10.3389/fendo.2022.1012244 (PMC9582268; doi:10.3389/fendo.2022.1012244)
Supplement: Supplementary file 1 [file DataSheet_1.docx]

Supplementary Material

# Search Strategy

exp Diabetes Mellitus, Type 2/

(type 2 adj5 diabetes).mp.

(type II adj5 diabetes).mp.

non-insulin dependent diabetes.mp.

T2DM.mp

NIDDM.mp

1 OR 2 OR 3 OR 4 OR 5 OR 6

exp Diabetes, Gestational/

gestational diabetes.mp.

pregnancy induced diabetes.mp.

pregnancy-induced diabetes.mp.

GDM.mp.

8 OR 9 OR 10 OR 11 OR 12

7 AND 13

Limit 14 to (english language and humans and yr= “2010-2021’’)

# Supplementary Figures and Tables

## Supplementary Tables

| **Supplemental Table 1 Study quality assessment using the Newcastle-Ottawa scale tool** | | | | | | | | | |
| --- | --- | --- | --- | --- | --- | --- | --- | --- | --- |
| **Study** | **Selection** | | | | **Comparability** | **Outcome** | | | **Total Stars** |
|  | **Representativeness of exposed cohort** | **Selection of non-exposed cohort** | **Ascertainment of exposure** | **Outcome of interest not present at start of study** | **Comparability of cohorts on the basis of the design or analysis** | **Assessment of outcome** | **Follow-up long enough for outcomes to occur** | **Adequacy of follow-up of cohorts** |  |
| **Hiersch et al., 2021** | ★ | ★ | ★ | ★ | ★ | ★ | ★ | ★ | 8 |
| **Wood et al., 2021** | ★ | ★ | ★ | ★ | - | ★ | ★ | ★ | 7 |
| **Bayoumi et al., 2021** | ★ | ★ | ★ | ★ | - | ★ | ★ | ★ | 7 |
| **Aziz et al., 2018** | ★ | ★ | ★ | ★ | - | ★ | ★ | - | 6 |
| **Lowe et al., 2018** | ★ | ★ | ★ | ★ | ★ | ★ | ★ | ★ | 8 |
| **Kugishima et al., 2018** | ★ | ★ | ★ | ★ | - | ★ | ★ | ★ | 7 |

## Supplementary Figures


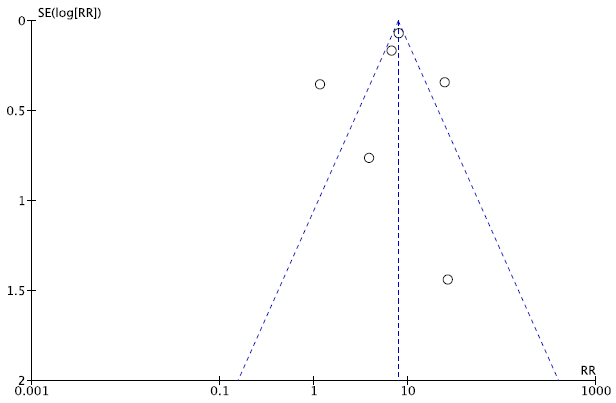


**Supplementary Figure 1.** Funnel plots for publication bias
